# Accessory Cavitated Uterine Malformation (ACUM): An Underdiagnosed, Treatable Cause of Dysmenorrhea

**DOI:** 10.7759/cureus.76650

**Published:** 2024-12-30

**Authors:** Vina Kumari, Mamta R Datta

**Affiliations:** 1 Department of Obstetrics and Gynaecology, Tata Main Hospital, Jamshedpur, IND

**Keywords:** acum, dysmenorrhea, gubernaculum, mri, mullerian duct

## Abstract

An uncommon and recently identified Müllerian anomaly is the accessory cavitated uterine mass (ACUM). It is distinguished by the presence of a noncommunicating auxiliary cavity inside the uterus, located near and surrounded by uterine smooth muscle, and bordered by functioning endometrium beneath the round ligament's insertion, with a perfectly healthy uterus, ovaries, tubes, and cavity.

Given that it is a congenital ailment with a persistent Müllerian duct at the level of the round ligament, primarily resulting from gubernaculum dysfunction, it usually manifests clinically as childhood dysmenorrhea in girls.

With a wide spectrum of differential diagnoses, including cystic adenomyoma, myoma, and adenomyosis, ACUM is an uncommon but treatable cause of severe dysmenorrhea and chronic pelvic pain in young girls.

While MRI is quite accurate in diagnosing ACUM, it requires a high level of suspicion due to various differential diagnoses, even though ultrasonography can identify ACUM with ease.

Similar entities have been misidentified or described in the past as adenomyoma (i.e., existence of histological organization similar to the uterus), cavitated uterus, and cystic adenomyoma (locally confined to myometrium), and myoma (resembles a lump inside the uterus). These are currently all believed to represent ACUM.

In this case study and retrospective analysis, we emphasize the unique clinical presentation and care of an imaging discovery of ACUM in a 16-year-old girl.

## Introduction

Accessory cavitated uterine mass (ACUM) is an uncommon and recently identified Müllerian anomaly. As no population-based studies have been conducted on ACUM, the exact prevalence of this recently identified entity is unknown. The uterus contains an accessory cavity, surrounded by uterine smooth muscle, and bordered by a functioning endometrium, along with a perfectly healthy uterus, ovaries, tubes, and cavity. The origin of this accessory cavity is embryological, primarily due to gubernaculum dysfunction. As a result of this dysfunction, the Müllerian duct is not reabsorbed completely and remains patent in the form of an accessory cavity, typically below the level of the round ligament insertion. Soon after menarche, menstrual blood starts collecting in the accessory cavity, and the patient typically presents with childhood dysmenorrhea. It has a wide spectrum of differential diagnoses, including cystic adenomyoma, myoma, and adenomyoma.

ACUM is an uncommon but treatable cause of severe dysmenorrhea and chronic pelvic pain in young girls. Ultrasound and MRI are both quite accurate in diagnosing ACUM but require a high level of suspicion due to various differential diagnoses. ACUM has been misdiagnosed in the past due to similar entities such as adenomyoma (a histological organization resembling the uterus), cavitated uterus, cystic adenomyoma (locally confined to the myometrium), and myoma (a lump inside the uterus). These are currently all believed to represent ACUM. In this case study and retrospective analysis, we emphasize the unique clinical presentation and care of the imaging discovery of ACUM in a 16-year-old girl.

## Case presentation

The labor room admitted a sixteen-year-old girl with acute dysmenorrhea. She needed intravenous analgesia to alleviate the pain because her severe menstrual cramps did not respond to oral medications, unlike the dysmenorrhea caused by fibroids or endometriosis. She reached menarche at the age of 14, and since then, she has been experiencing this cyclical pain. She consistently experienced severe dysmenorrhea, which interfered with her daily activities and did not respond to medication during her regular, three-day menstrual periods. Ultrasound results showed a normal-sized uterus with normal tubes and ovaries. We identified and diagnosed a 3 cm x 3 cm enlargement next to the left cornua as a fibroid, ruling out adenomyosis and a rudimentary horn as differential diagnoses.

**Figure 1:**
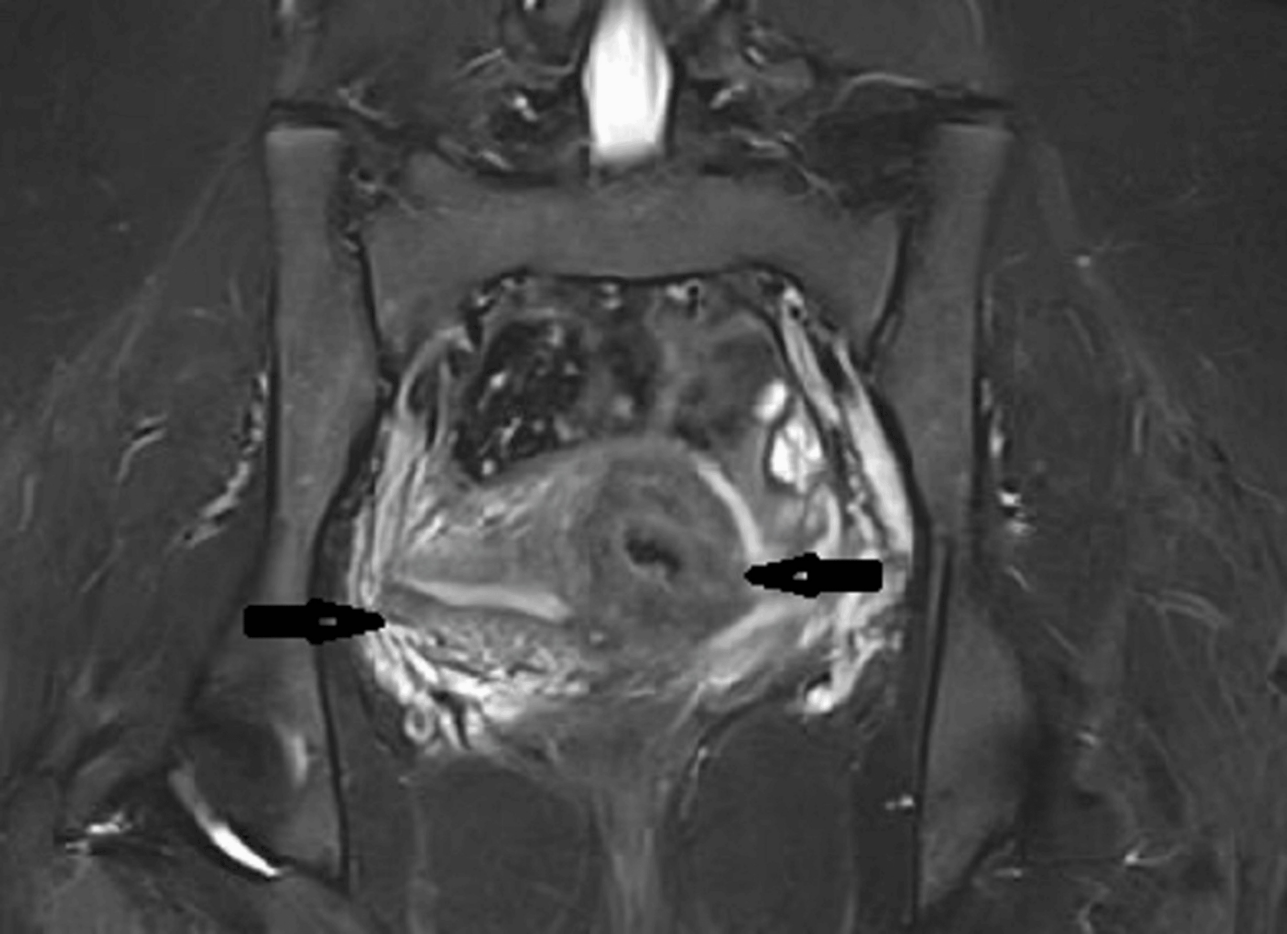
MRI showed a normal uterine cavity (arrow) with a distinct, round, noncommunicating cavitated lesion (arrow). A 3 cm x 4 cm mass is visible along the left anterior uterine wall, directly beneath the insertion of the round ligament. The T2 hyperintense endometrium lining the cavity contained hemorrhagic materials. On T1 images, it was hyperintense; on T2-weighted (T2W) images, it was hypointense.

Given the rarity of a fibroid manifesting at such a young age and causing severe dysmenorrhea, we conducted an MRI of the pelvis to better describe the adnexal mass. The MRI report showed a normal uterine cavity with a distinct, round, noncommunicating cavitated lesion. We observed a 3 cm x 4 cm mass along the left anterior uterine wall, directly beneath the insertion of the round ligament. The T2 hyperintense endometrium lining the cavity contained hemorrhagic materials. On T1 images, it was hyperintense; on T2-weighted images, it was hypointense (Figure 1).

**Figure 2:**
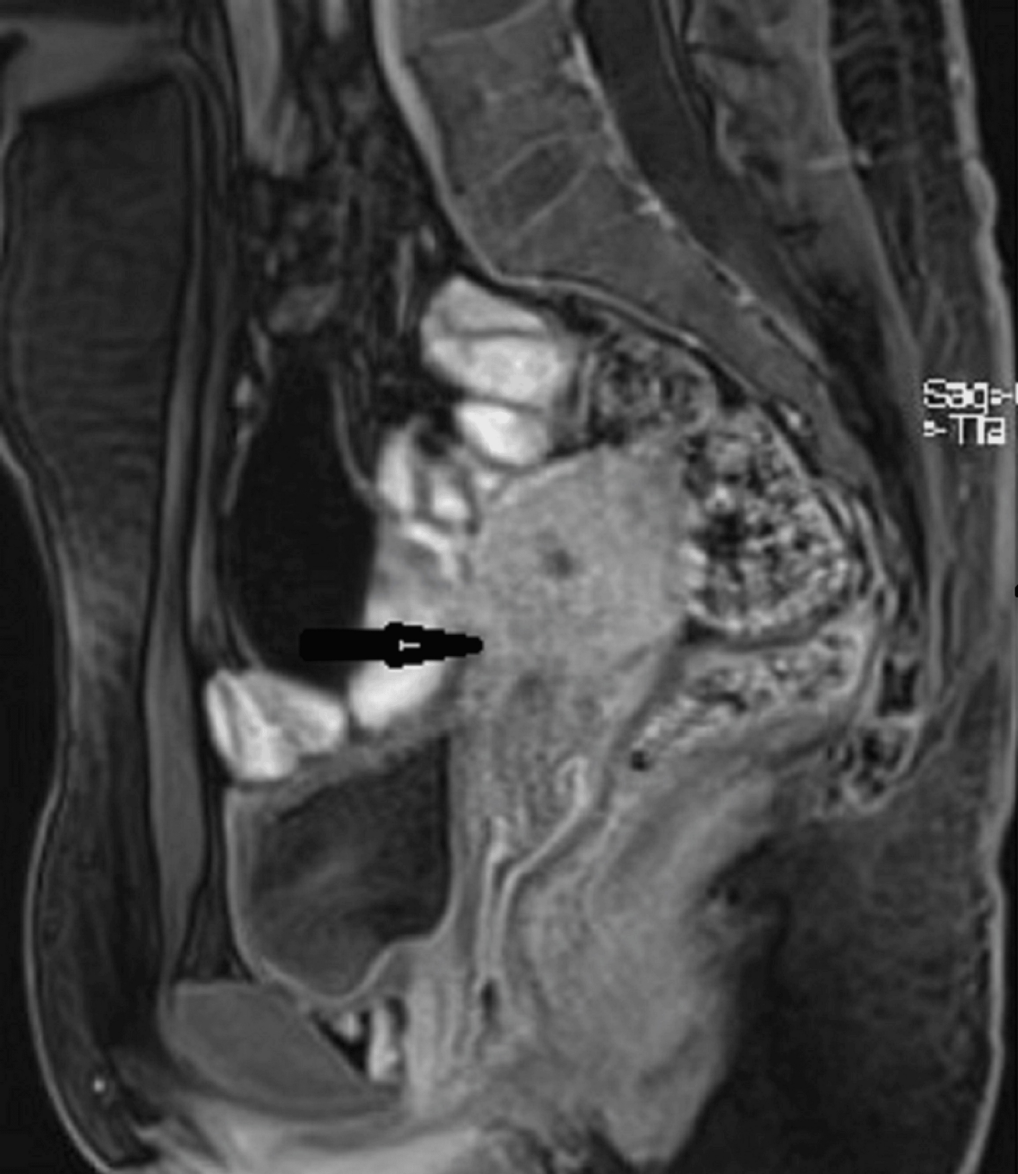
The junctional zone of the cavity thickened (13 mm), and the endomyometrial interface lacked clarity. The size and form of the major uterine cavity were normal, and the cornua were both clearly visible (arrow), ruling out the ultrasound diagnosis of a primitive horn.

The junctional zone of the cavity thickened (13 mm), and the endomyometrial interface lacked clarity. The size and form of the major uterine cavity were normal, and the cornua were both clearly visible. The USG diagnosis of a primitive horn was excluded in (Figure 2).

The major uterine cavity’s junctional zone, endomyometrial interface, and myometrial signal intensity were typical. The ovaries were both healthy. There was no hematosalpinx present, nor were there any signs of pelvic endometriotic deposits. The aforementioned information led to the consideration of ACUM as a diagnosis. Peyron N et al. provided a description of how MRI helped assess 11 patients with ACUM [1]. He concluded that MRI could aid in a prompt diagnosis due to its ability to identify well-defined lesions with a core hemorrhagic cavity, as well as help rule out other differential diagnoses. We explained the disease, its cause, and its treatment to the patient and her relatives. We discussed and decided that excision of the non-communicating cavity would be the definitive treatment for her severe dysmenorrhea.

Surgery (Intraoperatively)

**Figure 3:**
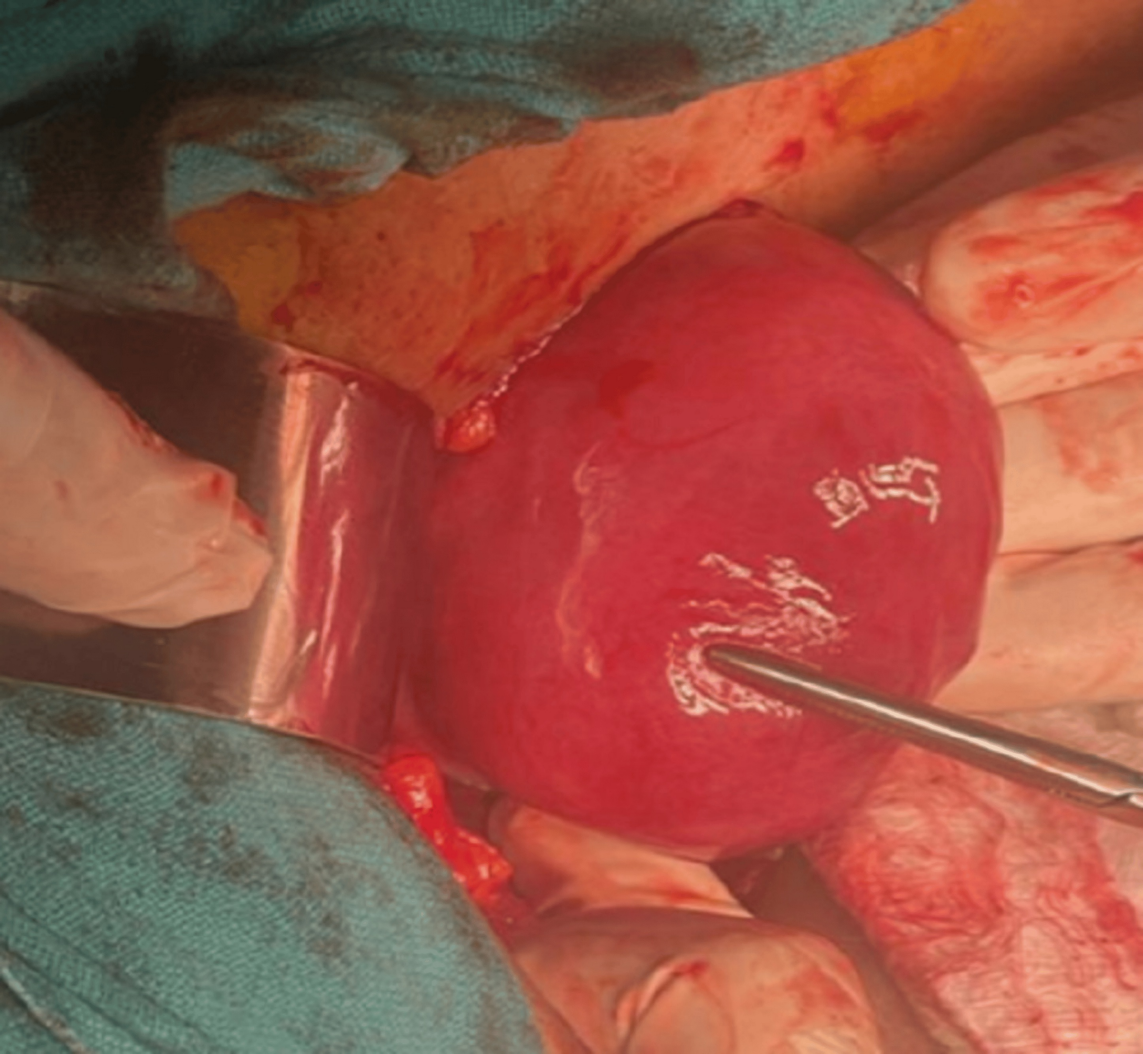
A 4 cm x 3 cm bulge was seen on the anterior surface of an absolutely normal-looking uterus (instrument pointer).

**Figure 4:**
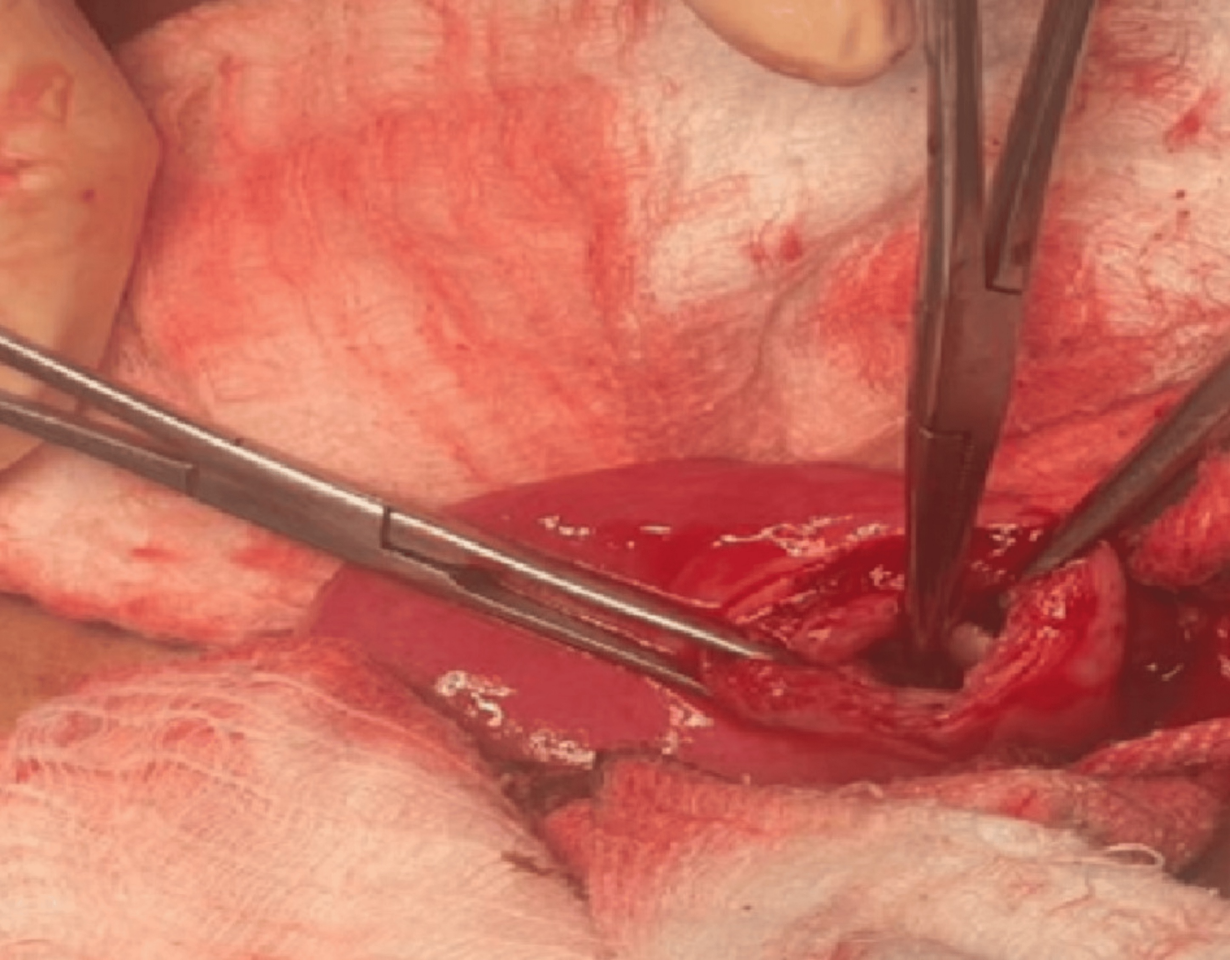
A transverse cut made over the bulge revealed an accessory cavity (instrument pointer) filled with chocolate-colored fluid, along with an absolutely normal, non-communicating uterine cavity.

We made a transverse cut across the mass's anterior wall (Figure 3) and extracted 10-12 milliliters of chocolate-colored fluid. We removed the functioning endometrium and its associated cavity, then sealed the defect. There was no connection with the main uterine cavity (Figure 4).

The patient's surgical course was unremarkable, and her symptoms progressively improved.

Histopathology (HPE)

**Figure 5:**
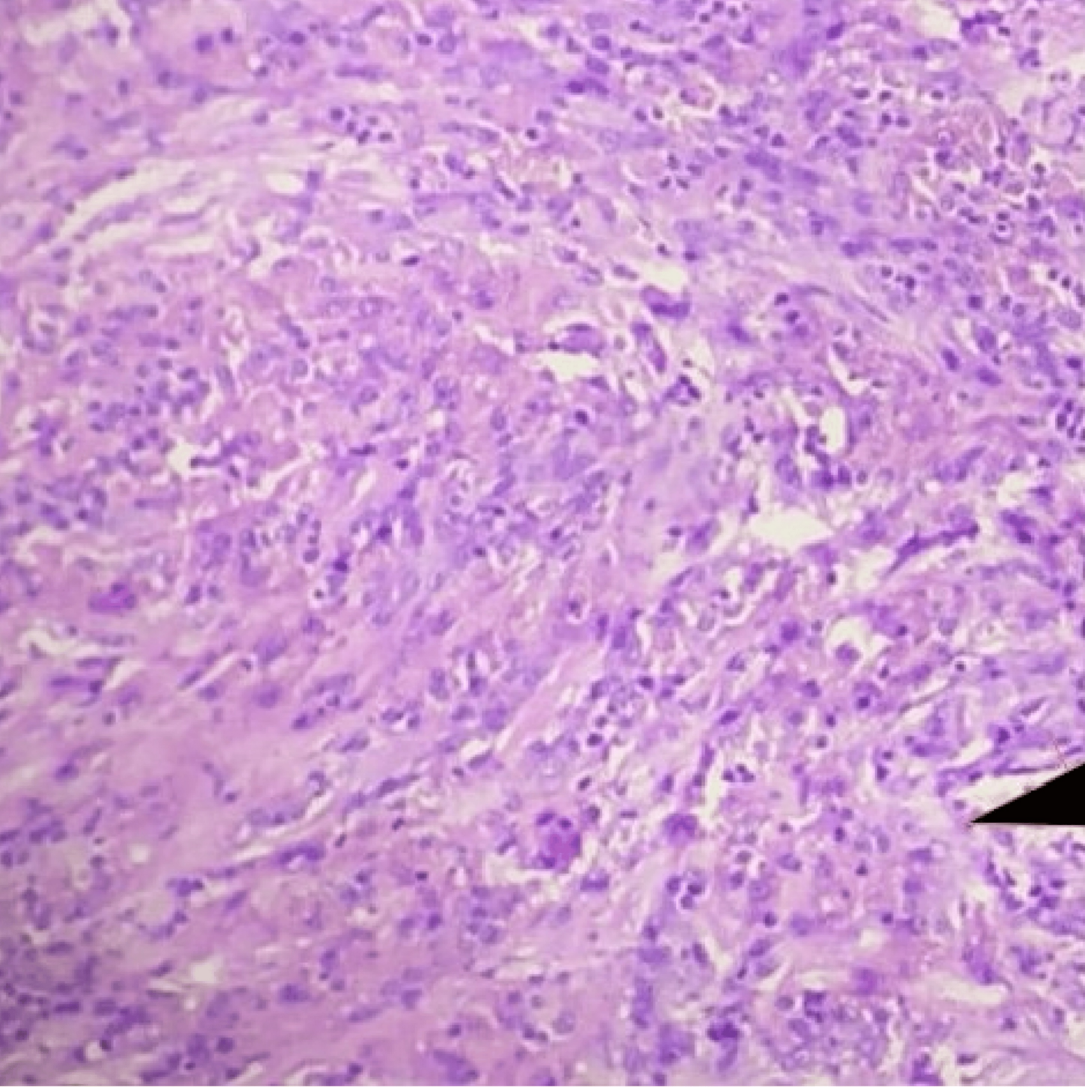
H&E-stained micrograph showing a hemosiderin-laden macrophage and inflammatory cells (arrow), magnification 10X.

**Figure 6:**
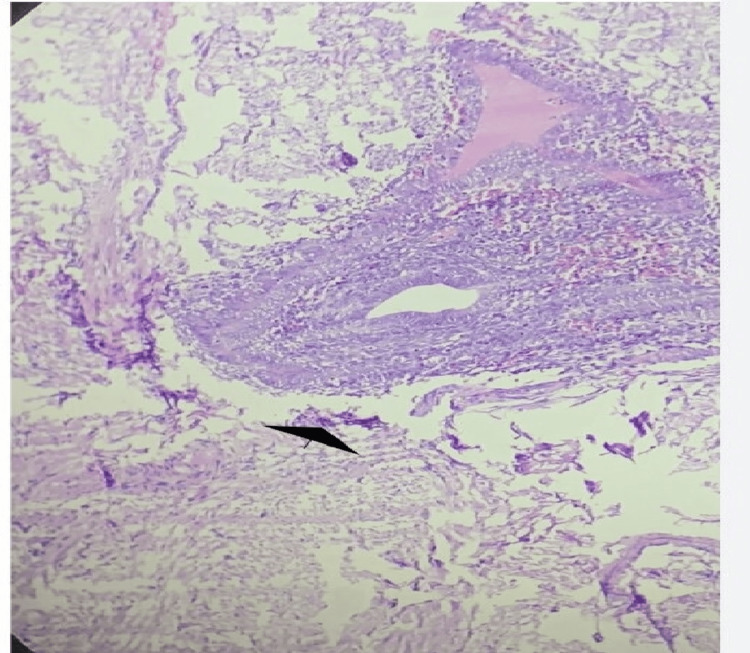
H&E stain microphotograph showing endometrial glands with compact stroma (arrow), magnification 40X.

Histopathology showed a cavitated mass surrounded by unevenly spaced smooth muscle cells and lined by functioning endometrium with glands and stroma. There were additional foci of adenomyosis in the myometrium of the tumor (Figures 5-6). In the smooth muscle cells, desmin, the estrogen receptor (ER), and the progesterone receptor (PR) all showed positive staining.

## Discussion

There have been other names for what we now refer to as ACUM, or Accessory Cavitated Uterine Malformation. These include uterine-like masses, solitary cystic adenomyomas, juvenile cystic adenomyosis, and accessory uterine cavities. In 2010, Acién P et al. examined eighteen cases that satisfied the suggested ACUM criteria [2]. The standards were based on histology, morphology, and location.

During the fifth and sixth week of development in the embryo, there exist mesonephric and paramesonephric ducts [3]. In the case of a female embryo, due to the absence of androgens and anti-Müllerian hormone, the paramesonephric or Müllerian duct completes its invagination and development. The gubernaculum (inguinal cone) grows over the Müllerian ducts and includes its muscular fibers. Lateral to this point, the Müllerian duct develops into the fallopian tube and medial to this into the normal uterus. The female gubernaculum itself develops into the uterine round ligament. This dysfunction in the female gubernaculum could potentially be the cause of the accessory cavity uterine malformation. All documented cases describe the distinctive placement of ACUM in the round ligament's insertion area or pathway, supporting the idea that maldevelopment of the gubernaculum causes ACUM [4-5].

Clinical presentation

Since the primary pathology of ACUM is the accumulation of menstrual fluid inside an enclosed cavity, most patients are under 30 years old and typically present with severe dysmenorrhea.

Investigation

Ultrasound, being the first line of investigation, the radiologist's knowledge of ultrasound features is especially crucial. ACUM is characterized by a distinctive echogenic fluid accumulation at one of the lateral aspects of the uterus, appearing as a marginated cavity within the myometrium. This might be mistaken for a fibroid or a rudimentary horn, which can be excluded by MRI.

MRI has been used for the final diagnosis in the majority of published case reviews and reports. As MRI uses all three imaging planes, both cornua are distinctively seen, ruling out Müllerian aberrations. According to Peyron N et al., MRI can detect ACUM as an accessory horn that is present in the broad ligament or external myometrium and is functional but noncommunicative [5]. ACUM features a characteristic distinct ring that resembles the junctional zone in terms of low T1, T2, and T3 signal enhancement. Other Müllerian anomalies are ruled out in the defined cases because of the presence of normal uterine cavities and no concurrent renal tract anomalies [6-7]. Hysterosalpingography and hysteroscopy are easy ways to determine that the uterus is normally constructed.

Histologically, ACUM resembles the uterus, featuring a central cavity encircled by a myometrial mantle and bordered by endometrium.

In the largest case series to date by Naftalin J et al. [8], the mean outer cavitary diameter of ACUM was reported to be 22.8 mm (95% CI: 20.9-24.8), and the mean interior cavitary diameter as 14.1 mm (95% CI: 12.2-16.1 mm). These measurements correspond to our patient's uterine mass parameters.

Rare as they are (about 7% of instances), reproductive system anomalies can cause severe dysmenorrhea in teenagers, as well as difficulties with fertility, such as infertility and recurrent spontaneous abortions in later life. Cases of ACUM have been reported in isolated case reports in the past; however, the diagnosis is either overlooked or misinterpreted as a degenerated fibroid or cystic adenomyoma because this congenital uterine malformation is not classified as a Müllerian anomaly by the European Society of Human Reproduction and Embryology. Also, there have been several reported cases of isolated cystic adenomyomas or masses that resemble a uterus with a similar auxiliary uterine chamber.

An accessory and cavitated uterine tumor, accompanied by a normally functioning endometrium, is a finding common to all case reports [9-12]. One of the diagnostic criteria for ACUM is the presence of glands and stroma in the accessory cavity lined with endometrial epithelium. Associated features include a functional endometrium in a normal uterus, surgical proof in the form of an excised mass and histology, chocolate-brown fluid content, and the lack of adenomyosis. In the case under discussion, each of these was present.

Dysmenorrhea in ACUM patients is not responsive to hormonal or analgesic drugs, and resection is the only appropriate therapy. Since patients are young and wish to maintain their fertility, resection of the mass without performing a hysterectomy is the best course of action. There have been no reports of recurrence of symptoms after resection.

In this case, the patient's symptoms fully resolved when the auxiliary cavity was removed, with substantial improvement in quality of life. During the six months that she was followed up, she did not experience any severe episodes of spasmodic dysmenorrhea and did not take a single day off from school.

## Conclusions

The number of published case reports on ACUM is only 13. Cystic adenomyoma is the most common misdiagnosis of ACUM. Unlike cystic adenomyomas, which do have endometrial glands and stroma, ACUM also contains uterine tissue and receptors. When diagnosing ACUM, factors such as the patient's age, the location of the gubernaculum defect, the onset of symptoms, and the frequency of recurring episodes of the ailment should all be considered. MRI is highly accurate in making the diagnosis. The preoperative diagnosis of ACUM should be suggested by MRI findings of an accessory cavitated uterine localized mass located below the attachment of the round ligament, usually with hemorrhagic contents, an otherwise normal-shaped uterus with both cornua identified normally, without any evidence of adenomyosis, and bilateral normal tubes and ovaries.
